# Case Report: Eyebrow pilonidal sinus with bridging scar

**DOI:** 10.3389/fsurg.2026.1838726

**Published:** 2026-06-24

**Authors:** Yanpeng Zhu, Xuemei Tao, Xiaoliang Yang, Ruiyun Wang, Ke Xue

**Affiliations:** 1 Plastic and Aesthetic Outpatient Clinic, Qingdao Central Hospital, University of Health and Rehabilitation Sciences, Qingdao, Shandong, China; 2 Department of Hyperbaric Oxygen, Qingdao Eighth People's Hospital, Qingdao, Shandong, China; 3 Department of Burns and Plastic Surgery, Qingdao Central Hospital, University of Health and Rehabilitation Sciences, Qingdao, Shandong, China; 4 Department of Plastic Surgery, Rizhao Hospital of Traditional Chinese Medicine, Rizhao, Shandong, China; 5 Department of Plastic Surgery, Pudong Gongli Hospital, Shanghai University of Medicine & Health Sciences, Shanghai, China; 6 Department of Burn and Plastic Surgery, Hainan Western Central Hospital, Danzhou, Hainan, China

**Keywords:** bridging scar, case report, eyebrow, pilonidal sinus, plastic surgery

## Abstract

**Background:**

Pilonidal sinus is an acquired inflammatory disease associated with trauma and subcutaneous hair implantation, most commonly occurring in the sacrococcygeal region. Facial pilonidal sinus is extremely rare, with only about 10 cases reported globally, all localized to the cheek, and none with associated bridging scar formation.

**Case presentation:**

We report the first case of an eyebrow pilonidal sinus with a 29-year disease course accompanied by ipsilateral bridging scar formation. A 56-year-old man presented with right eyebrow swelling and intermittent drainage for 29 years following accidental trauma during high school. Physical examination revealed subcutaneous induration and epidermal depigmentation of the forehead and glabella, along with a bridging scar between the right eyebrow and nasal root. The patient complained of a sensation of temporal visual field obstruction due to mechanical traction. Surgical excision with adjacent flap transfer was performed initially, followed by forehead tissue expander placement and Z-plasty revision for recurrent scarring. Three sessions of postoperative intralesional injections (triamcinolone acetonide + fluorouracil) were administered as a prophylactic measure. Pathology confirmed chronic post-traumatic pilonidal sinus with foreign body giant cell reaction. At 1-year follow-up, the scar, visual field, and appearance were significantly improved with no recurrence.

**Conclusion:**

This first reported case of eyebrow pilonidal sinus with bridging scar highlights the importance of considering this rare entity in the differential diagnosis of chronic facial lesions. Staged surgical reconstruction combined with adjuvant therapy can achieve satisfactory functional and aesthetic outcomes in this challenging anatomical area.

## 1 Introduction

Pilonidal sinus is an acquired inflammatory disease characterized by chronic infection of the subcutaneous tissue containing hair, typically occurring in the sacrococcygeal region of young, hirsute, obese males, with a global incidence of 26/100,000 (1, 2). While atypical sites such as the axilla, umbilicus, perineum, scalp, interdigital spaces, and nasal root (14) account for only 2.2% of cases (2–4), facial involvement is exceptionally rare. As of 2025, only slightly over 10 cases of facial pilonidal sinus have been reported worldwide, all localized to the cheek region (2, 5–8). Most of these cases were associated with frequent shaving, supporting the theory of traumatic hair implantation as the primary etiology (9–11). Importantly, none of the reported facial cases documented associated bridging scar formation.

Bridging scars, also known as trapdoor or tunnel scars, represent a unique form of cicatricial malunion where a strip of relatively normal skin bridges across a scarred area, typically resulting from chronic inflammatory destruction of the dermal base with relative preservation of the overlying epidermis. The eyebrow region is particularly challenging for surgical reconstruction due to its complex musculature (frontalis, corrugator supercilii, procerus, orbicularis oculi) and aesthetic significance.

Herein, we report the first case of pilonidal sinus occurring in the eyebrow region with a 29-year disease course, uniquely complicated by ipsilateral bridging scar formation across the medial canthus. This case highlights the importance of considering pilonidal sinus in the differential diagnosis of chronic inflammatory facial lesions and demonstrates a staged surgical approach for functional and aesthetic reconstruction in this challenging anatomical area.

## 2 Case report

A 56-year-old man of Asian ethnicity presented to our clinic with a 29-year history of right eyebrow swelling and intermittent drainage. At age 17 during high school, he accidentally struck his right eyebrow against a desk corner. The initial trauma caused no skin break, but local swelling developed within weeks, followed by intermittent serous drainage that persisted for years without medical treatment. Over time, a raised, progressively depigmented mass appeared in the right eyebrow region. Concurrently, the adjacent forehead, medial upper eyelid, glabella, and medial canthal area became gradually indurated.

The patient had no history of systemic diseases (diabetes, hypertension, autoimmune disorders), no family history of similar conditions, and no history of recurrent infections elsewhere. He denied any history of shaving the eyebrow area or any prior surgical procedures.

**Figure 1:**
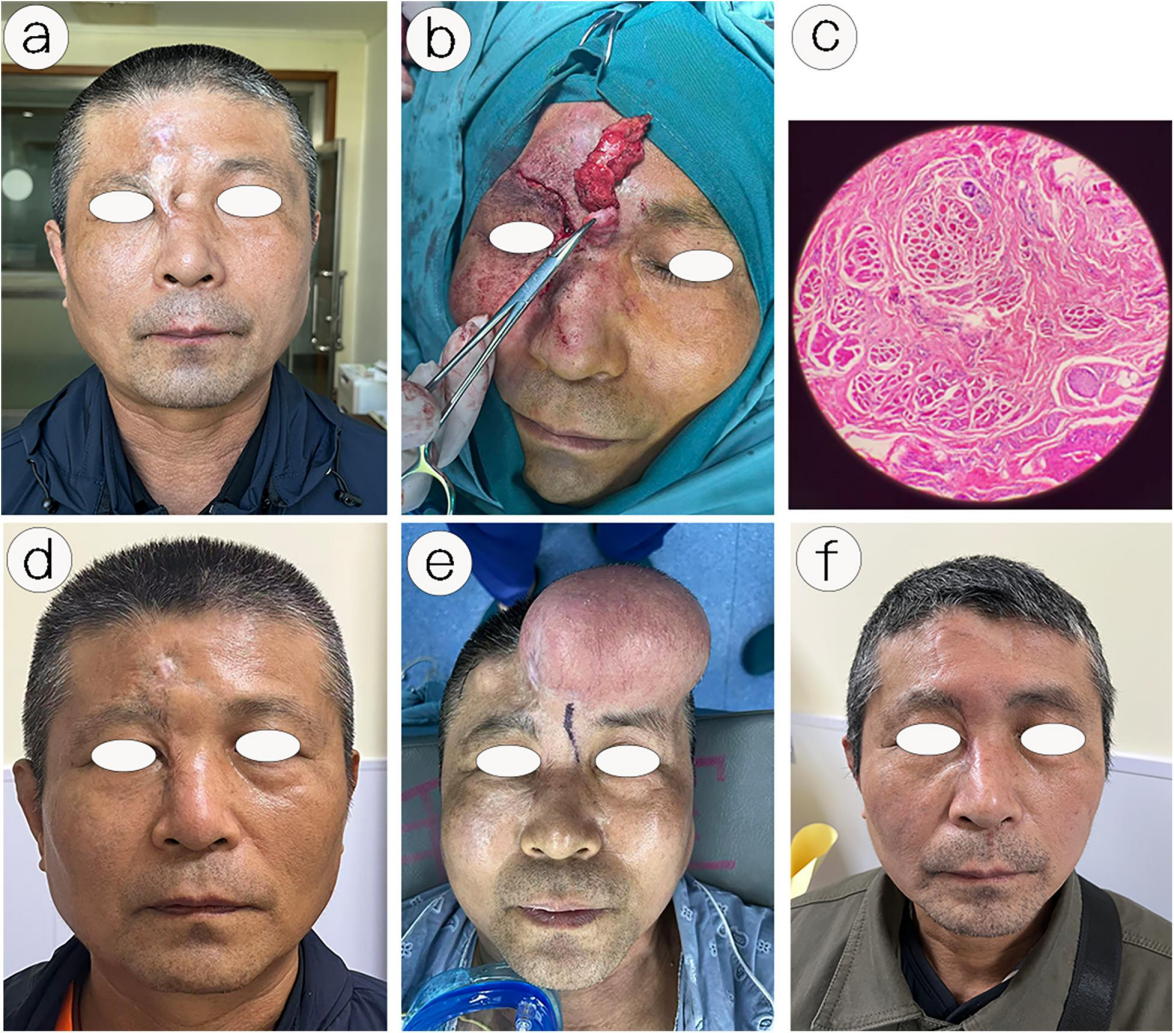
**(A)** preoperative view showing the bridging scar between the right eyebrow and nasal root, with depigmentation of the forehead and glabella. **(B)** Intraoperative view during the first surgery demonstrating excision of the glabellar and nasal root scars with adjacent flap transfer. **(C)** Histopathological examination revealing hyalinized connective tissue, skeletal muscle, adipose tissue, and hyperplastic nerve fibers arranged haphazardly, with foreign body giant cell reaction (HE staining, × 100). **(D)** Postoperative view at one-year follow-up showing significant improvement in scar appearance, visual field, and overall facial aesthetics with no evidence of recurrence.

Physical examination revealed subcutaneous induration and epidermal depigmentation of the right forehead and glabella. A distinct bridging scar extended from the right eyebrow to the right nasal root, with a 4 mm strip of normal skin overlying the medial canthus spared (Figure 1A). The bridging scar was approximately 2.5 cm in length, elevated above the surrounding skin surface, with a tunnel-like appearance. The right medial canthus morphology and monocular vision were normal; however, the patient complained of a sensation of temporal visual field obstruction due to mechanical traction of the scar tissue, affecting his quality of life. No palpable lymphadenopathy was present.

The preoperative differential diagnosis included epidermal inclusion cyst, dermoid cyst, pilomatrixoma, post-traumatic scar, and foreign body granuloma. Organized hematoma with secondary infection, soft tissue calcification, or ossification were also considered but ruled out by the absence of relevant imaging findings and histopathological evidence. While a pilonidal sinus was suspected based on clinical history, a definitive diagnosis was only confirmed postoperatively by histopathology.

Under local anesthesia, the patient underwent excision of the glabellar and nasal root scars with adjacent flap revision and transfer (Figure 1B). Forehead scar excision was deferred due to insufficient local flap availability. Intraoperatively, subcutaneous granulation tissue with hyperplastic, sclerotic scar tissue was identified. Within the hyperplastic tissue beneath the eyebrow, fine eyebrow hairs were embedded, with no purulent fluid present. The excised specimen measured 4.5 × 2.5 × 0.8 cm.

Postoperative pathological examination revealed hyalinized connective tissue, skeletal muscle, adipose tissue, and hyperplastic nerve fibers arranged haphazardly, with foreign body giant cell reaction to keratinous material (Figure 1C). No epithelial lining or malignant cells were identified. Based on the trauma history, intraoperative findings of embedded hairs, and pathological evidence of foreign body reaction to hair fragments, a definitive diagnosis of chronic post-traumatic pilonidal sinus with bridging scar formation was established. This definitive diagnosis, confirmed after the first surgery, informed our second-stage reconstructive strategy, confirming that the primary disease had been completely excised and we were managing a residual scar.

Postoperatively, the visual field obstruction improved significantly. The patient reported a clear subjective improvement in visual field expansion after surgery. However, at 3-month follow-up, scarring recurred in the glabellar and nasal areas, causing traction on the nose and eyebrow. The forehead scar also remained unresolved. Therefore, four months after the first surgery, a 50 mL forehead tissue expander was implanted under local anesthesia, followed by weekly saline inflation over 4 months. Five months after expander placement, under general anesthesia, the expander was removed, the forehead scar was excised with advancement flap transfer, and Z-plasty revision of the recurrent nasal scar was performed (Table 1). Three sessions of postoperative intralesional injections (triamcinolone acetonide 40 mg + 2.5% fluorouracil 0.6 mL + 2% lidocaine 4 mL) were administered at monthly intervals. These injections were administered as a prophylactic measure to prevent excessive scar formation, rather than as a treatment for established keloid or hypertrophic scar.

**Table 1:** Summary of Key revisions.

Location	Original Text	Revised Text
First surgery	On June 18, 2024 (first surgery)	Under local anesthesia
Second surgery	on October 24, 2024 (second surgery)	four months after the first surgery
Third surgery	On March 4, 2025 (five months after expander placement, third surgery)	Five months after expander placement
Follow-up	(March 2026)	Removed (implied by “At 1-year follow-up”)

At 1-year follow-up (Figure 1D), the scar appearance was significantly improved with minimal residual scarring. The visual field was completely restored subjectively, and overall facial aesthetics were satisfactory. The patient reported no pain, itching, or recurrence of drainage. There was no evidence of recurrence on clinical examination. Given the potential for late recurrence reported in sacrococcygeal pilonidal sinus, continued follow-up at 6-month intervals is planned for the next 2 years (12). The patient expressed high satisfaction with both the functional and aesthetic outcomes of the staged surgical approach.

## 3 Discussion

Pilonidal sinus is traditionally considered a disease of the sacrococcygeal region, with atypical sites accounting for only 2.2% of cases (2–4). Facial involvement is exceptionally rare, with only 12 cases reported in the English literature to date (5–8, 10). All previously reported facial cases were localized to the cheek region and associated with frequent shaving in males, supporting the acquired theory of traumatic hair implantation (9–11). None of these cases described bridging scar formation, making our case unique in two aspects: the eyebrow location has never been reported, and bridging scar across the medial canthus represents a previously undocumented phenomenon.

### 3.1 Pathogenesis of bridging scar formation

Based on the 29-year disease course and sequential clinical observations, we postulate three stages: (1) Pilonidal sinus formation: trauma implanted eyebrow hairs into the dermis, establishing a localized chronic foreign body inflammatory reaction; (2) Progressive dermal destruction: persistent low-grade inflammation gradually eroded the surrounding dermis while the overlying epidermis remained relatively intact; (3) Bridging scar formation: during the post-inflammatory healing phase, scar tissue formed in the destroyed dermal areas, while the preserved epidermal strips created the characteristic “bridge” appearance. The relative resistance of the epidermis to inflammatory destruction compared to the dermis is well recognized, as the epidermis functions as both a physical and immunological barrier that actively protects against pathogens and environmental threats (13).

The centrifugal subcutaneous extension of inflammation in our case, resembling keloidal “crab-claw” invasion, may be explained by two factors: gravitational effects (the eyebrow region is relatively elevated compared to the medial canthus and nasal root, facilitating dependent tracking of inflammatory exudate) and anatomical factors (the high mobility of the medial canthal skin may direct inflammation along deeper planes rather than superficial erosion).

### 3.2 Surgical considerations and alternative strategies

Surgical management of pilonidal sinus depends on disease stage and location (12, 15, 16). For chronic-phase facial lesions, complete excision with primary reconstruction is recommended to minimize recurrence and optimize aesthetic outcomes (5). Our case highlights two challenges specific to the eyebrow region: limited local tissue availability for primary closure necessitated staged reconstruction with tissue expansion, and the unique muscular environment (frontalis, corrugator supercilii, procerus, orbicularis oculi) predisposes to recurrent scar hyperplasia despite complete excision.

An alternative surgical strategy would be placing a tissue expander in the frontal region at the initial stage, followed by lesion excision and simultaneous flap advancement in a second stage. While this approach offers the advantage of a single reconstructive procedure, it risks placing an expander beneath an actively inflamed field, potentially increasing infection and expander extrusion rates. Our staged approach—lesion excision first, followed by delayed expansion and reconstruction after inflammation subsided—provided a safer alternative in this chronic inflammatory setting.

### 3.3 Strengths and limitations

The primary strength of this report is that it documents the first case of eyebrow pilonidal sinus with associated bridging scar formation, providing unique insight into the natural history of this condition over a 29-year period. The staged surgical approach with tissue expansion and adjuvant therapy offers a potential strategy for similar cases, though this observation requires validation in larger case series.

Limitations inherent to a single case report include limited generalizability. Long-term follow-up beyond 1 year is ongoing to monitor for late recurrence, which has been reported in sacrococcygeal pilonidal sinus. Additionally, the visual field improvement was assessed subjectively by the patient rather than through quantitative ophthalmological testing, which would strengthen future reports.

### 3.4 Take-away lessons

Pilonidal sinus should be considered in the differential diagnosis of chronic inflammatory lesions in hair-bearing facial areas, even in the absence of shaving history. Bridging scar formation may result from chronic inflammatory destruction of the dermis with relative preservation of the epidermis. Staged surgical reconstruction with tissue expansion and adjuvant intralesional therapy appears promising for achieving satisfactory outcomes in this challenging anatomical region. Complete excision with pathological confirmation is essential for definitive diagnosis and to exclude malignancy.

## 4 Conclusion

This first reported case of eyebrow pilonidal sinus with bridging scar expands the clinical spectrum of atypical pilonidal sinus and highlights the importance of considering this entity in the differential diagnosis of chronic inflammatory facial lesions. Staged surgical reconstruction with tissue expansion and adjuvant intralesional therapy can achieve satisfactory functional and aesthetic outcomes in this challenging anatomical location.

## Data Availability

The original contributions presented in the study are included in the article/Supplementary Material, further inquiries can be directed to the corresponding author/s.
